# Network-Based Comparative Analysis of *Arabidopsis* Immune Responses to *Golovinomyces orontii* and *Botrytis cinerea* Infections

**DOI:** 10.1038/srep19149

**Published:** 2016-01-11

**Authors:** Zhenhong Jiang, Xiaobao Dong, Ziding Zhang

**Affiliations:** 1 State Key Laboratory of Agrobiotechnology, College of Biological Sciences, China Agricultural University, Beijing 100193, China

## Abstract

A comprehensive exploration of common and specific plant responses to biotrophs and necrotrophs is necessary for a better understanding of plant immunity. Here, we compared the *Arabidopsis* defense responses evoked by the biotrophic fungus *Golovinomyces orontii* and the necrotrophic fungus *Botrytis cinerea* through integrative network analysis. Two time-course transcriptional datasets were integrated with an *Arabidopsis* protein-protein interaction (PPI) network to construct a *G. orontii* conditional PPI sub-network (gCPIN) and a *B. cinerea* conditional PPI sub-network (bCPIN). We found that hubs in gCPIN and bCPIN played important roles in disease resistance. Hubs in bCPIN evolved faster than hubs in gCPIN, indicating the different selection pressures imposed on plants by different pathogens. By analyzing the common network from gCPIN and bCPIN, we identified two network components in which the genes were heavily involved in defense and development, respectively. The co-expression relationships between interacting proteins connecting the two components were different under *G. orontii* and *B. cinerea* infection conditions. Closer inspection revealed that auxin-related genes were overrepresented in the interactions connecting these two components, suggesting a critical role of auxin signaling in regulating the different co-expression relationships. Our work may provide new insights into plant defense responses against pathogens with different lifestyles.

Plant pathogens, including viruses, bacteria, fungi, oomycetes and nematodes, can cause severe economic and ecological damage. According to their lifestyles, plant pathogens can be generally divided into two major categories, biotrophs and necrotrophs. Biotrophs feed on living host cells. Thus, they keep host cells alive during their invasion to complete their life cycles. Powdery mildew is a fungal disease that affects a wide range of plant species, including many economically important crops^1^. As a powdery mildew fungus, *Golovinomyces orontii* has an obligate biotrophic lifestyle, and it has been shown to colonize *Arabidopsis* under controlled laboratory conditions^2^. In contrast, necrotrophs acquire their nutrients from dead cells. Necrotrophs often secrete enzymes and plant toxins into host cells to kill and degrade them^3^. *Botrytis cinerea* is recognized as a typical necrotrophic fungus that causes grey mould disease^4^. *B. cinerea* affects over 200 crop species, resulting in serious economic losses. The life cycles of *G. orontii* and *B. cinerea* on *Arabidopsis* follow a defined infection progression, including conidium germination, appressorium formation, penetration of the host surface and conidiophore formation^2^,^3^. Under optimal conditions, the infection cycles of *G. orontii* and *B. cinerea* require approximately 5 and 3–4 days, respectively.

A multitude of studies have investigated plant defense responses against pathogens with different lifestyles, making great contributions to our understanding of plant immunity^5^,^6^,^7^. In particular, it is well established that plant hormones, such as salicylic acid (SA), jasmonic acid (JA) and ethylene (ET), play a central role in the regulation of plant immune responses. However, their functional roles differ in plant immune responses against pathogens with different lifestyles^5^,^8^,^9^,^10^. SA has been shown to induce defense against biotrophs, whereas JA/ET positively mediates immunity to necrotrophs^8^. For example, mutants in SA biosynthesis or signal transduction are more susceptible to *G. orontii*^11^,^12^. Both an ET-insensitive mutant (*ein2-1*) and a JA-insensitive mutant (*coi1-1*) have been reported to be highly susceptible to *B. cinerea* infection, which demonstrates the important roles of JA and ET in resisting *B. cinerea*^13^,^14^. In addition, plant defense responses usually result in reduced plant growth^15^. It has been clearly demonstrated that plant growth hormones, such as auxin, gibberellin (GA) and brassinosteroid (BR), regulate the trade-off between plant growth and immunity^16^,^17^,^18^. As a key regulator of plant growth and development, auxin is heavily involved in regulating plant immunity^19^. *Arabidopsis* mutants with repressed auxin signaling show increased resistance to the biotrophic pathogen *Pseudomonas syringae* but increased susceptibility to the necrotrophic pathogen *B. cinerea*^20^. The plant hormone GA promotes plant growth and development by degrading DELLA proteins, a type of growth-repressing protein^21^. DELLA proteins have also been found to promote disease susceptibility in biotrophs and resistance to necrotrophs by modulating JA and SA signaling^22^. Recent studies have also highlighted the role of the BR signaling pathway in plant immunity. For instance, the BR-activated transcription factor (TF) BZR1 (for BRASSINAZOLE-RESISTANT 1) has been reported to mediate plant growth and immunity by interacting with several WRKY TFs^23^.

Recently, high-throughput experiments have resulted in the increasing availability of omics data (e.g., interactomes and transcriptomes). The availability of these data for plant stress responses provides a good opportunity to employ computational systems biology approaches to advance our understanding of plant stress responses. For example, a meta-analysis of 386 *Arabidopsis* microarray samples was conducted to detect genes and co-expression modules common to drought and bacterial stress responses^24^. By a comparative analysis of differentially expressed genes responding to *P. syringae* infection or attack by the insect *Brevicoryne brassicae*, Barah *et al.* explored the general and attacker-specific defense response genes in *Arabidopsis*^25^. In 2014, Tully *et al.* employed the concept of biological networks to better interpret immune-related transcriptomic data^26^. They generated a genome-wide *Arabidopsis* immune co-expression network using large-scale transcriptional data and identified 156 distinct immune-related functional modules. Recently, we also employed an advanced machine learning method to integrate the *Arabidopsis* gene network with a series of transcriptional data^27^. Through comprehensive network analysis, we revealed shared and distinct plant gene network organizations between pattern-triggered immunity and effector-triggered immunity.

Although many experimental studies have been carried out to decipher general plant immune responses, a systematic analysis that integrates different omics data has not been used to compare plant defense responses to pathogens with different lifestyles. Recently, microarray experiments measuring plant immune responses to the biotrophic pathogen *G. orontii* and necrotrophic pathogen *B. cinerea* have been conducted^28^, providing important data resources for further computational analyses. In this work, we conducted a comparative analysis of plant defense responses to *G. orontii* and *B. cinerea* by integrating transcriptional data and the *Arabidopsis* PPI network (Fig. 1a). By mapping time-course transcriptional data to PPIs, we constructed two conditional PPI sub-networks, namely the *G. orontii* conditional PPI sub-network (gCPIN) and the *B. cinerea* conditional PPI sub-network (bCPIN), to characterize the plant defense responses against *G. orontii* and *B. cinerea*. First, we assessed the biological significance of the two conditional PPI sub-networks and focused on the analysis of hub proteins in plant immunity. Moreover, by comparing the two conditional PPI sub-networks, we were able to reveal two network components that were involved in plant development and defense, respectively. We attempted to explain the distinct expression correlations between interacting proteins connecting the two network components during plant defense response to pathogens with different lifestyles. Finally, we developed a website for the scientific community to interactively explore the networks constructed in our work.

## Results and Discussion

### Experimental PPI Network and Time-Course Transcriptional Data Sets in *Arabidopsis* Immune Responses

Experimental *Arabidopsis* PPI data were collected from three publicly available molecular interaction databases, TAIR^29^, IntAct^30^ and BioGRID^31^. Predicted PPIs were not considered in our work due to their relatively low reliability. Thus, we obtained 6,640 proteins and 16,797 experimentally validated interactions, which constituted the primary PPI network in this work.

Two series of time-course transcriptional data, which measured the transcriptional responses of *Arabidopsis* to two different pathogens, were used in our work. The first (GEO accession number: GSE5686) was generated by the AtGenExpress project, which detected *Arabidopsis* defense responses at 8 different time points during infection by a biotrophic fungus (i.e., *G. orontii*). The second (GEO accession number: GSE29642) was produced by Windram *et al.* and contained 24 time points after inoculation with a necrotrophic fungus (i.e., *B. cinerea*)^28^.

We removed proteins without expression values in either transcriptional dataset from the primary PPI network. The retained network (AraPPINet), covering 5,598 proteins and 13,328 interactions, was used for the further construction of conditional PPI sub-networks (Supplemental Table S1).

### Construction of PPI Sub-networks Responding to Different Pathogens

As an important strategy to integrate the transcriptome data and PPI network, gene expression correlations between interacting proteins have been widely used to identify conditional sub-networks^32^,^33^,^34^. By only considering PPIs with high gene expression correlation, such integration is able to detect condition-specific protein interactions. To construct the conditional PPI sub-networks under *G. orontii* or *B. cinerea* infection (i.e., gCPIN and bCPIN), the corresponding transcriptional data were integrated into AraPPINet. The Pearson correlation coefficient (PCC) was employed to measure the gene expression correlation between two interacting proteins. Transcriptional data from infected tissues were used to calculate the PCC value for each interaction. The biological significance of a PCC value depends on the corresponding transcriptional data and the choice of normalization method^35^. To obtain a significant PCC threshold for each transcriptional data, we chose the PCC threshold based on the random PCC distribution (see Materials and Methods for details). First, we randomly permuted the transcriptional data and generated a random PCC distribution based on the permuted data. Then, the PCC value ranked in the top 10% from the random PCC distribution was selected as the threshold. Thus, the threshold values of 0.27 and 0.50 were selected for the construction of gCPIN and bCPIN, respectively. Keeping interactions with PCC above the threshold, we obtained two conditional PPI sub-networks: gCPIN, including 4,353 interactions between 3,101 proteins; and bCPIN, covering 3,388 proteins and 4,615 interactions (Supplemental Fig. S1 and Supplemental Table S1).

**Figure 1:**
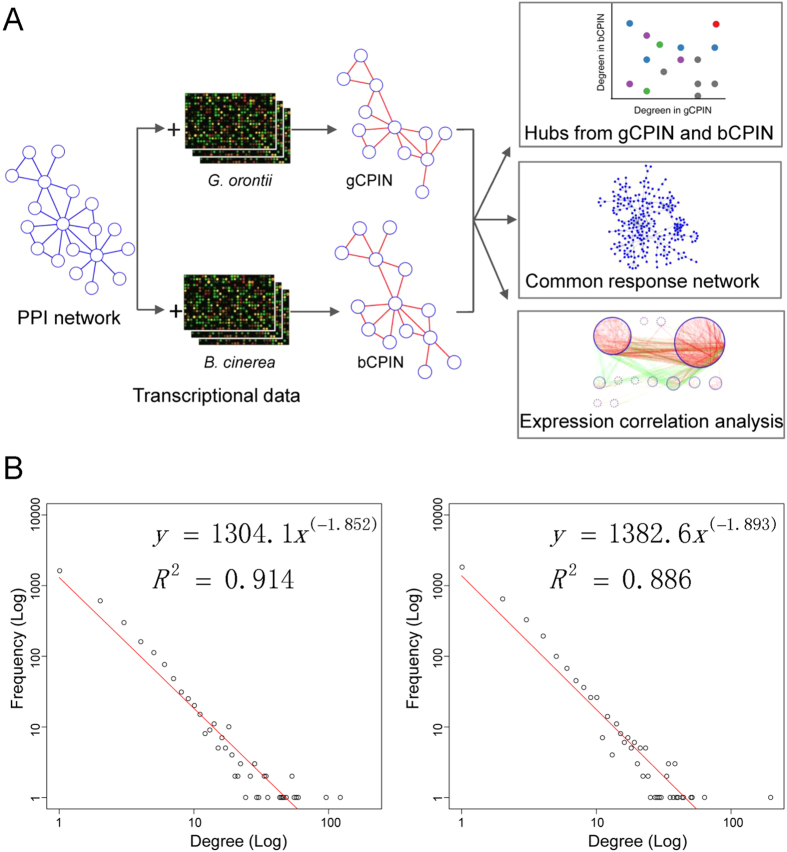
Construction of conditional PPI sub-networks reflecting *Arabidopsis* immune responses to *G. orontii* and *B. cinerea*. (**a**) We integrated time-course transcriptional data into a static *Arabidopsis* PPI network to obtain two conditional PPI sub-networks by keeping positively correlated interactions with PCC values larger than the given thresholds. The PCC thresholds of 0.27 and 0.50 were chosen for gCPIN and bCPIN, respectively. We first compared the hubs from gCPIN and bCPIN. Then, we constructed a common response network by selecting common edges from gCPIN and bCPIN. Finally, we measured the relationship between network components from the common response network by expression correlation analysis. (**b**) Degree distribution of gCPIN (left) and bCPIN (right). Frequency-degree relationship is plotted on a logarithmic scale. Degree (x) represents the number of edges for each node, and frequency (y) measures the number of nodes with a given degree. Both gCPIN and bCPIN follow a power-law distribution.

To assess the biological significance of gCPIN and bCPIN, a series of analyses were carried out, including topological analysis, modularity analysis and functional enrichment analysis. Several global network topological parameters that reflect the general arrangement of nodes or interactions within gCPIN and bCPIN are displayed in Supplemental Table S2. Generally, gCPIN and bCPIN displayed topological properties similar to typical biological networks. For instance, the node degrees in the two conditional PPI sub-networks followed the power-law distribution^36^ (Fig. 1b). Moreover, most biological networks can also be organized into modules, which are defined as clusters of closely connected nodes inside a network^37^,^38^. In this work, the Markov Cluster Algorithm (MCL) algorithm was used to identify network modules from the two conditional PPI sub-networks^39^. In total, we obtained 364 and 380 modules from gCPIN and bCPIN, respectively. For gCPIN and bCPIN, approximately 85% and 84% proteins were represented in modules, indicating that our conditional PPI sub-networks were organized into modules, similarly to most biological networks^40^. Gene annotation analysis showed that most modules could be enriched with at least one Gene Ontology (GO) term. For the 364 modules from gCPIN, 295 were enriched for at least one GO term. For the 380 modules from bCPIN, 315 were enriched for at least one GO term. Among these modules with enriched GO terms, 37 modules from gPCIN and 44 modules from bPCIN were annotated with the GO term “defense responses”. Many modules from gCPIN or bCPIN were also annotated with plant hormone (SA, JA or ET)-related GO terms (Supplemental Table S3). Moreover, we manually collected 538 plant defense-related genes (see Materials and Methods). By mapping the 538 genes to AraPPINet, gCPIN and bCPIN, we found that plant defense-related genes were significantly enriched in gCPIN (hypergeometric test, *p*-value = 2.49 × 10^−4^) and bCPIN (hypergeometric test, *p*-value = 0.0429) compared to AraPPINet.

Taken together, these results indicated the biological significance of gCPIN and bCPIN. In the subsequent analysis, we focused on the comparative analysis between gCPIN and bCPIN for the investigation of plant defense responses to *G. orontii* and *B. cinerea*.

### Hubs Play Important Roles in Plant Immunity

In a PPI network, hubs are generally defined as proteins (nodes) with a significantly higher degree than other nodes^41^. In this work, the top 10% of highly connected proteins in the two conditional PPI sub-networks were selected as hubs. We identified 418 hubs in gCPIN and 407 hubs in bCPIN. To examine their functional roles in plant immunity, we analyzed the enrichment of plant defense-related genes, TFs and hormone-related genes in hubs. As expected, plant defense-related genes were significantly enriched in hubs from gCPIN and bCPIN (hypergeometric test, *p*-value = 5.98 × 10^−6^ and 7.18 × 10^−5^, respectively). Plant hormones and TFs have been reported to play vital roles in plant immunity^8^,^42^. In total, we collected 1,231 plant hormone-related genes from AHD2.0^43^, covering eight hormone types [i.e., auxin, abscisic acid (ABA), GA, cytokinin (CK), ET, BR, SA and JA] and 1,717 TFs from PlantTFDB^44^ (see Materials and Methods). Similarly, plant hormone-related genes and TFs were overrepresented in hubs (statistical *p*-values are listed in Supplemental Table S4).

**Figure 2:**
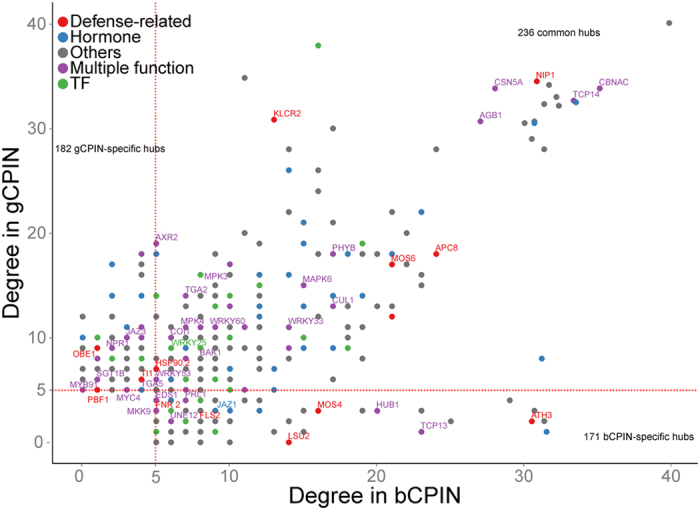
Scatter plot of hub proteins. The degree of hub proteins in bCPIN is plotted against the corresponding degree in gCPIN. For better presentation, hubs with degrees larger than 30 were normalized to the degree region between 30 and 40. The functional roles of hubs in hormone signaling, transcriptional regulation or plant immunity are displayed using different colors. Red, blue and green nodes represent defense-related genes, hormone-related genes and TFs, respectively. Hubs with multiple functional roles are colored in purple. The remaining hubs are colored in grey. Plant defense-related hubs are marked with their symbols. Because many nodes have the same degrees in two sub-networks, they are overlapped in the scatter plot. For better presentation, a web-based scatter plot is also available at http://systbio.cau.edu.cn/BN/index.php.

We divided the hubs in gCPIN and bCPIN into three groups (gCPIN-specific hubs, bCPIN-specific hubs and common hubs). The gCPIN-specific hubs were hubs only in gCPIN, the bCPIN-specific hubs were hubs only in bCPIN, and common hubs were hubs shared by bCPIN and gCPIN. We obtained 182 gCPIN-specific hubs, 171-bCPIN specific hubs and 236 common hubs (Fig. 2). The distinct roles of these condition-specific hubs in the plant defense responses to biotrophs and necrotrophs should be strongly related to their corresponding interaction partners in gCPIN and bCPIN. For example, SA receptor NPR1 (for NONEXPRESSOR OF PATHOGENESIS-RELATED PROTEINS1) had 9 interaction partners in gCPIN and 2 partners in bCPIN (Supplemental Fig. S2A), which was consistent with the important role of SA signaling in resisting biotrophic pathogens. For the nine interaction partners of NPR1 in gCPIN, four were TGA (for TGACG sequence-specific binding protein) transcriptional factors (TGA1, TGA2, TGA3 and TGA7). Interactions between NPR1 and TGAs can further regulate the expression of *PATHOGENESIS-RELATED* genes that confer resistance to pathogens^45^. The *Arabidopsis* RING E3 ligase, HUB1 (for HISTONE MONOUBIQUITINATION1) was shown to be essential for resistance to *B. cinerea,* while *hub1* mutant plants exhibited no effect on resistance to *P. syringae*^46^. In our conditional PPI sub-networks, HUB1 had 20 interaction partners in bCPIN and only 3 partners in gCPIN (Supplemental Fig. S2B). It is reasonable to speculate that HUB1 regulates plant immunity to *B. cinerea* through wide interactions with its partners in bCPIN. To further decipher the molecular mechanism of HUB1 in regulating plant immunity to necrotrophs, these partners can serve as important candidates for experimental verification. More details regarding hub degree distribution can be interactively explored through our website (http://systbio.cau.edu.cn/BN/index.php).

### Hubs in bCPIN Evolve Faster than Hubs in gCPIN

**Figure 3:**
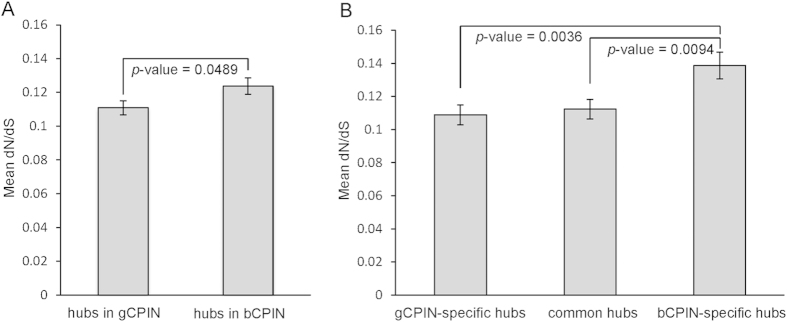
Hubs in gCPIN are more evolutionarily constrained than hubs in bCPIN. (**a**) Mean dN/dS (±s.e.) for two classes of hubs. Hubs in gCPIN and bCPIN both experience purifying selection with low mean dN/dS values. However, hubs in gCPIN are more evolutionarily constrained than hubs in bCPIN, with a significant *p*-value < 0.05 (Student’s *t* test). (**b**) Mean dN/dS (±s.e.) for gCPIN-specific hubs, bCPIN-specific hubs and common hubs. The results show that bCPIN-specific hubs have higher dN/dS than gCPIN-specific hubs (Student’s *t* test, *p*-value < 0.01) and common hubs (Student’s *t* test, *p*-value < 0.01).

We also investigated the selection pressures imposed on hubs by pathogens with different lifestyles. For this purpose, we analyzed the evolutionary rates of hubs by calculating their dN/dS (i.e., the ratio between nonsynonymous and synonymous substitution rates) values between *Arabidopsis* and *Carica papaya* (see Materials and Methods). We found that the average dN/dS values of hubs in both gCPIN and bCPIN were much smaller than 1, which indicated that the hubs had experienced strong purifying selection (Fig. 3a). This result is not surprising, as the node connectivity in a PPI network is negatively correlated with evolution rate^47^. Comparatively, the average dN/dS ratio of hubs in gCPIN was significantly lower than hubs in bCPIN, indicating that hubs in bCPIN evolved faster than hubs in gCPIN. We further compared the evolutionary rates of three groups of hubs defined previously (gCPIN-specific hubs, bCPIN-specific hubs and common hubs). As expected, the average dN/dS ratio of bCPIN-specific hubs was significantly higher compared to gCPIN-specific and common hubs (Fig. 3b). Considering that the two conditional PPI sub-networks were composed of interactions responding to *G. orontii* and *B. cinerea* infection, these results indicate the different selection pressures imposed on plants by pathogens with different lifestyles. Compared with *G. orontii*, the damage imposed by the necrotrophic pathogen *B. cinerea* on the plant was more destructive. Therefore, hubs involved in the response to *B. cinerea* invasion are likely to evolve faster to ensure successful defense.

### Common Response Network from gCPIN and bCPIN

To further investigate the relationship between plant immune responses induced by *G. orontii* and *B. cinerea*, we selected common edges from gCPIN and bCPIN and constructed a common response network covering 1,702 nodes and 1,619 edges (Supplemental Table S1 and Supplemental Fig. S3). GO annotation of the common response network showed that many biological processes in *Arabidopsis* were influenced by both *G. orontii* and *B. cinerea* (Supplemental Fig. S4). Approximately 35% of nodes (517 of 1,478) were annotated to the term response to stimulus with an adjusted *p*-value of 9.20 × 10^−92^ (hypergeometric test with Benjamini-Hochberg correction), which was consistent with the biological significance of the common response network. Developmental processes were also prominently enriched in the common response network, and the corrected *p*-value for this term was 9.47 × 10^−46^, which was consistent with the common knowledge that plant growth and development are influenced during the plant immune response^48^. Other biological processes, such as photosynthesis and vesicle-mediated transport, were also enriched. Detailed descriptions of all the enriched GO terms are provided in Supplemental Table S5.

**Figure 4:**
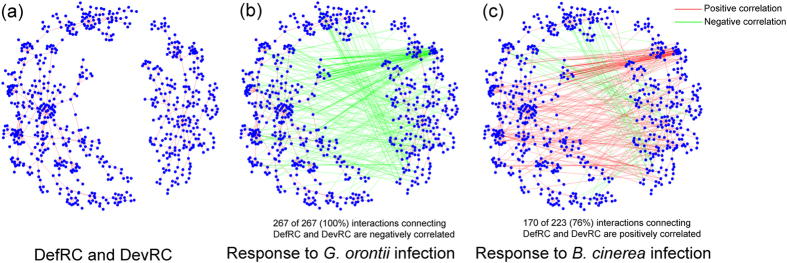
Different expression correlations between DevRC and DefRC. Nodes represent proteins, red edges represent positively correlated interactions between two nodes, and green edges represent negatively correlated interactions. (**a**) Interactions in two connected components, DefRC (left) and DevRC (right), are all positively correlated under *G. orontii* and *B. cinerea* infection conditions. (**b**) Co-expression relationship calculated using time-course transcriptional data from *G. orontii*-infected tissues. (**c**) Co-expression relationships calculated using time-course transcriptional data from *B. cinerea*-infected tissues.

In terms of network organization, the common response network was organized into many components (Supplemental Fig. S3). A component was defined as a group of connected nodes disconnected from the other nodes in the network. We extracted 109 components with at least three proteins from the common response network, many of which were enriched with GO terms. For example, we found that the two largest components were significantly enriched in plant development and plant defense responses (Table 1; Full annotations of these two components are listed in Supplemental Table S5). The largest component, consisting of 468 nodes and 560 edges, was annotated as “defense response” with a significant *p*-value of 5.47 × 10^−13^. The second largest component, consisting of 258 nodes and 330 edges, was significantly enriched with “developmental process” (*p*-value = 1.06 × 10^−13^). According to their biological functions, we named the two components DefRC (Defense-Related Component) and DevRC (Development-Related Component) (Fig. 4a).

To investigate whether the identification of components was related to the choice of PCC thresholds, we reconstructed and analyzed the common response network using a more stringent cutoff (Supplemental Fig. S5A). The results showed that the identification of these components was largely stable with the alteration of PCC threshold, although the resulting components were smaller with the stricter threshold. Moreover, we permuted the transcriptional data by shuffling the expression values for each gene among different time points. The result showed that the components constructed from our method cannot be generated using the randomized data (Supplemental Fig. S5B). Most nodes of the common response network resulting from randomized data were discrete or organized into extremely small components. The above control experiments clearly demonstrate that these components are mainly determined by the transcriptome data obtained from tissues infected by *G. orontii* and *B. cinerea*.

### Distinct Expression Correlation of Interactions Connecting Network Components

To better understand the relationships of the network components, we connected the network components using interactions from AraPPINet, which were further classified according to the expression correlation under *G. orontii* or *B. cinerea* infection conditions. We found that interactions connecting sizable network components (containing at least 10 proteins) often had different expression correlations under different conditions (Supplemental Fig. S6). Interactions between some components were positively correlated under *G. orontii* infection condition but negatively correlated under *B. cinerea* infection condition, and vice versa. The expression correlation between DevRC and DefRC showed the most significant difference. There were 325 interactions between DevRC and DefRC in AraPPINet. For simplicity, we divided these interactions into positive and negative interactions using the PCC values calculated from the transcriptome data. Positive interactions were defined the same way as in the construction of the conditional PPI sub-network. To define negative interactions, we selected a threshold corresponding to the 10% lowest PCCs in the random PCC distribution, and a negative interaction was assigned if the PCC value was below the threshold (See Materials and Methods). Thresholds of −0.5 and −0.27 were then selected to define negatively correlated interactions under *G. orontii* and *B. cinerea* infection conditions, respectively. Under *G. orontii* infection condition, 267 interactions connecting DevRC and DefRC were negatively correlated (Fig. 4b), and there were no positively correlated interactions. By contrast, we obtained 170 positive interactions and 53 negative interactions between DevRC and DefRC based on *B. cinerea* responsive transcriptional data (Fig. 4c).

In addition to the distinct expression correlation between DevRC and DefRC, the expression patterns of genes in DevRC and DefRC were also different in response to *G. orontii* and *B. cinerea* infections. Here, a gene was defined as differentially expressed if its expression was altered by greater than 1.2-fold change between spore-infected (treatment) and mock-treated (control) plants at any time point. All genes in DefRC and DevRC were differentially expressed following *B. cinerea* infection. Similarly, after *G. orontii* infection, most genes in DefRC (88.7%) and DevRC (87.2%) were differentially expressed. The extensive changes in gene expression in DefRC and DevRC further showed the activation of plant defenses and the impact on plant development following *G. orontii* and *B. cinerea* infection.

We also compared the number of differentially expressed genes in DevRC and DefRC at different stages of infection. According to the infection cycle, time-course transcriptional data were divided into three stages (see Materials and Methods). Following *G. orontii* infection, many genes in DefRC were up-regulated at all three stages, which revealed the activation of plant immunity (Supplemental Fig. S7A). After *B. cinerea* infection, many genes were also up-regulated in DefRC. However, more genes were suppressed, and the number of suppressed genes increased with infection stage (Supplemental Fig. S7B). *B. cinerea* is an aggressive necrotrophic pathogen that secretes enzymes and toxic molecules to kill host cells. Therefore, along with the activation, partial defense responses were suppressed, and the suppression got stronger as the infection progressed. Regarding DevRC, more genes tended to be down-regulated over the course of *G. orontii* or *B. cinerea* infection. This result is consistent with many previous findings that plants suppress developmental processes to allocate energy to resist pathogen invasion^49^,^50^,^51^. The repression of plant development was stronger under *B. cinerea* infection (Supplemental Fig. S7C,D). At the early stage of *B. cinerea* infection, more than half (55.8%) of DevRC genes were suppressed, and almost all genes (95.3%) were suppressed at the late stage.

### Enrichment of Auxin-Related Genes on the Dev-Def Interface

The interface connecting two components is the place where different biological processes coordinate with each other^32^,^33^ and deserves further investigation. The Dev-Def interface was defined as interactions connecting DevRC and DefRC. We obtained 325 PPIs among 240 genes on the Dev-Def interface. We found that some genes on the Dev-Def interface were already known to participate in the regulation of plant growth and defense (Table 2). Moreover, more than 60% genes on Dev-Def interface were hubs in gCPIN or bCPIN, further indicating their important roles.

We noticed that auxin-related genes were overrepresented on the Dev-Def interface, which may better explain how the plant regulates the trade-off between growth and immunity. For the 38 auxin-related genes in DevRC and DefRC, 30 genes were distributed on the Dev-Def interface (hypergeometric test, *p*-value = 3.20 × 10^−9^). Auxin is known to regulate many aspects of plant growth and development. Its role in plant-pathogen interaction has also been widely reported^19^. Some auxin-related genes on the interface, such as *ARF1* (for *AUXIN RESPONSE FACTOR 1*), *ARF2* (for *AUXIN RESPONSE FACTOR 2*) and *AXR6* (for *AUXIN RESISTANT 6*) (Table 2), have been documented as involved in plant immunity. Moreover, other hormone genes regulating the trade-off between plant growth and immunity also appeared on the interface (Table 2). For example, *BZR1*, a positive regulator of the BR signaling pathway, was recently identified as an important regulator mediating the trade-off between plant growth and immunity^23^.

We further examined the interactions between 30 auxin-related genes located on the Dev-Def interface. Similar to the differential expression correlation of interactions between DevRC and DefRC, all interactions between the 30 auxin-related genes were negatively correlated in response to *G. orontii* infection but positively correlated in response to *B. cinerea* infection (Supplemental Fig. S8). Previous studies have demonstrated that auxin signaling is essential for necrotrophic resistance but induces susceptibility to biotrophs^20^,^52^. In agreement with the above finding, the different correlations of interactions between auxin-related Dev-Def interface proteins may provide new insights into the functional roles of auxin-related genes in the regulation of plant immune responses against different lifestyle pathogens.

### A Web Tool for Interactive Network Visualization

For the convenience of the research community, we have created a user-friendly website to interactively explore and visualize the networks constructed in our study (http://systbio.cau.edu.cn/BN/index.php). The website was implemented using Sigmajs Exporter, a plugin in Gephi (https://marketplace.gephi.org/plugin/sigmajs-exporter/). In addition to the interactive network exploration, we also converted the scatterplot of hubs in gCPIN and bCPIN into an interactive web application based on Shiny (http://shiny.rstudio.com/).

### Limitations of Current Work

Our results are based on currently available interactome and transcriptional data and must be interpreted with caution. One major limitation of the comparative analysis is the bias of the expression data used to construct gCPIN and bCPIN. For example, the transcriptional data GSE5686 were collected from only leaves 7–10, while the transcriptional data GSE29642 were collected from leaf 7. We conducted a computational experiment to investigate how the conditional PPI sub-network can be affected when different gene expression data measuring the same pathogen infection were used. For this purpose, we constructed a new gCPIN (gCPIN-GSE13739) using another dataset, GSE13739, which measures *Arabidopsis* gene expression at 6 time points after inoculation with *G. orontii*^53^. Using the same workflow as the construction of the original gCPIN, the resulting gCPIN-GSE13739 covered 2,754 nodes and 3,454 edges. The number of shared PPIs between gCPIN-GSE13739 and the original gCPIN was 2,266 (the proportion of overlapping interactions was 65.6%). We also permutated the expression data of GSE5686 1,000 times and constructed a conditional network for each permutation. The average number of overlapping interactions between randomly conditional networks and gCPIN was 482 (the average proportion of overlapping interactions was 35.1%). In general, the fraction of PPIs shared by gCPIN and gCPIN-GSE13739 was significantly higher than the fraction shared by gCPIN and 1,000 randomly conditional networks (Supplemental Fig. S9, Student’s *t* test *p*-value < 2.20 × 10^–16^). This significant overlap indicated that the construction of gCPIN can capture the core PPIs related to the infection of *G. orontii*, but we also observed a large number of different PPIs between the two gCPINs. Thus, it is possible that the results of the comparative analysis would also be affected by the use of different expression data. To obtain more reliable results, therefore, using expression data under identical laboratory conditions and treatments would be a better choice.

Another limitation of the current work is that we only considered highly correlated PPIs in the construction of gCPIN and bCPIN. On the one hand, the available *Arabidopsis* interactome is still far from complete. Some genes without interaction partners in the current coverage are not included in this work, but they might play important functional roles in plant immunity. On the other hand, less abundant or tissue-specific transcripts may be missed by retaining only PPIs with high expression correlation. Through analysing the expression levels of genes from AraPPINet and two conditional PPI sub-networks, we found that the expression levels of genes from gCPIN or bCPIN were significantly higher than the expression levels of genes from AraPPINet (Supplemental Fig. S10, Student’s *t* test, *p*-value = 3.12 × 10^–5^ for gCPIN vs. AraPPINet and *p*-value = 6.79 × 10^–6^ for gCPIN vs. AraPPINet). Moreover, we also downloaded 746 tissue-specific *Arabidopsis* genes from the literature^54^. Of these 746 tissue-specific genes, 109 appeared on AraPPINet. After data integration, 63 of these 109 genes were not included in gCPIN (hypergeometric test, *p*-value = 3.55 × 10^–3^), and 60 genes were excluded from bCPIN (hypergeometric test, *p*-value = 6.43 × 10^–4^). The above analyses showed that less abundant or tissue-specific genes tended to be filtered out by our method. Undoubtedly, the *Arabidopsis* interactome will become more complete, and more time-course transcriptional data measuring *Arabidopsis* gene expression under pathogen infection will be generated in the near future. The availability of these data will allow scientists to design more advanced workflows, perform more comprehensive analyses and obtain more reliable results.

## Conclusion

In summary, we constructed two conditional PPI sub-networks (gCPIN and bCPIN) to compare plant immune responses against the biotrophic pathogen *G. orontii* and the necrotrophic pathogen *B. cinerea* by integrating transcriptional data and *Arabidopsis* PPI data. First, we found that hubs in gCPIN and bPCIN played important functional roles in plant immunity. Plant defense-related genes, plant hormone-related genes and TFs were overrepresented in hubs; the distinct roles of gCPIN/bPCIN-specific hubs in plant defense responses to biotrophs and necrotrophs should be related to their different interaction partners in two networks. Moreover, we found that hubs in bCPIN evolved faster than hubs in gCPIN. By analyzing common interactions from gPCIN and bCPIN, we further identified two major network components (DefRC and DevRC), in which the defense responses and development processes were enriched, respectively. Interestingly, the gene expression relationship between DefRC and DevRC was positively correlated under *B. cinerea* infection condition but negatively correlated under *G. orontii* infection condition. Several proteins involved in the interactions connecting DefRC and DevRC were found to participate in the regulation of the trade-off between plant immunity and development. Finally, we noted an enrichment of auxin-related proteins involved in the interactions connecting DefRC and DevRC, which might explain the distinct relationships between DefRC and DevRC under different conditions. Taken together, we hope that the current comparative analysis on plant immune responses to pathogens with different lifestyles will help to improve our systems understanding of plant immunity.

## Materials and Methods

### Data Collection and Preprocessing

Two series of transcriptional data were downloaded from the NCBI Gene Expression Omnibus (GEO), and the corresponding GEO accession numbers were GSE5686 and GSE29642^55^. The raw data of GSE5686 were normalized using the Bioconductor R package affy^56^. Repeated samples for GSE5686 were averaged to obtain the final expression value. For GSE29642, we directly downloaded the normalized data from the GEO database. For the two normalized gene transcriptional datasets, probe sets were mapped to their corresponding gene symbols, and the expression values of replicated probes of the same gene symbol were averaged. To assemble the protein-protein interaction network, 16,797 experimentally verified PPIs covering 6,640 different proteins were collected from the TAIR Interactome 2.0^29^, IntAct^30^ and BioGRID^31^. *Arabidopsis* TFs were downloaded from the Plant Transcription Factor Database (PlantTFDB), which is a public database devoted to identifying and categorizing all plant genes involved in transcriptional control^44^. *Arabidopsis* hormone-related genes, which are defined as genes participating in the biosynthesis, metabolism, transport, perception or signaling pathways of plant hormones, were gathered from the *Arabidopsis* Hormone Database 2.0 (AHD2.0)^43^.

Plant defense-related genes were collected in two ways. The major way was analyzing gene ontology annotation^57^. We downloaded GO annotation files for *Arabidopsis* from the FTP site of TAIR (ftp://ftp.arabidopsis.org/home/tair/Ontologies/). Then, for each record in the annotation file, if the description of a gene met the following two criteria, we selected the gene as a plant defense-related gene. First, the record should use experimental evidence codes, including Inferred from Experiment (EXP), Inferred from Direct Assay (IDA), Inferred from Physical Interaction (IPI), Inferred from Mutant Phenotype (IMP), Inferred from Genetic Interaction (IGI), and Inferred from Expression Pattern (IEP). Second, the GO term should contain biological process keywords, including “systemic acquired resistance”, “systemic resistance”, “immune” and “defense response to fungus”. The other method used to collect plant defense-related genes was literature retrieval. We first searched the literature in PubMed with the keywords “*Botrytis cinerea*” and “powdery mildew”; then, we selected plant defense-related genes through literature reading.

### Determination of PCC Threshold

Many methods exist to determine a PCC threshold, including the use of an arbitrary cutoff 58, keeping the top 1% of correlations^59^, and determining cutoffs based on correlation graph structure^60^,^61^ or statistical significance^62^. To keep biologically relevant interactions, we set different cutoffs for the two sets of transcriptional data. First, we employed PCC to measure expression correlations between two interacting proteins. PCC was calculated by the following formula:


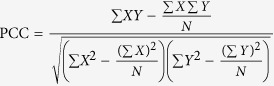


where *X* and *Y* are the log-transferred gene expression values of two interacting proteins, and *N* represents the number of time points in the corresponding transcriptional data. Then, we randomly permuted transcriptional data for the 5,598 proteins across different time points and calculated PCCs for each permutation to achieve a random PCC distribution. Finally, we sorted the random PCC distribution and chose the PCC value at the end of the top 10% highest (lowest) PCCs as the threshold of positively (negatively) correlated interactions.

### Network Visualization and Analysis

Cytoscape (version 3.0.2) and its plugins were employed to visualize the network constructed in this study and perform network analysis^63^. The Cytoscape plugin Network Analyzer was used to conduct network topological analysis and extract connected components. Network modules from the two conditional PPI sub-networks were identified using the MCL package (http://micans.org/mcl/), an implementation of the MCL algorithm^39^. Compared with other existing clustering algorithms, MCL is superior for the extraction of complexes from interaction networks^64^. The recommended inflation parameter of 1.8 was adopted. Modules with fewer than three proteins were discarded from our analysis.

### Evaluation of Evolutionary Rate

To estimate the evolutionary rate of hubs, we compared orthologous sequences between *Arabidopsis* and *C. papaya*. First, we downloaded the protein sequences and coding sequences (CDS) of *Arabidopsis* and *C. papaya* from the PLAZA database^65^. Then, the InParanoid algorithm (http://inparanoid.sbc.su.se/cgi-bin/index.cgi) was used to identify the orthologs of *Arabidopsis* hub proteins in *C. papaya*. Finally, for each pair of orthologs, we calculated dN/dS using the yn00 program in the PAML package^66^,^67^. The dN/dS ratio is employed to infer the direction and magnitude of natural selection acting on protein-coding genes. A dN/dS ratio of 1.0 indicates neutral evolution, a lower ratio (dN/dS < 1.0) indicates purifying (negative) selection, and a higher ratio (dN/dS > 1.0) indicates positive selection.

### Differential Expression Analysis

A gene was identified as differentially expressed if its expression value exhibited a greater than 1.2-fold change between the spore-infected (treatment) and mock-treated (control) plants at any time point. Normalized transcriptional data were used to identify differential expression genes. For better comparisons, the two groups of time-course transcriptional data were divided into three stages based on the life cycles of *G. orontii* and *B. cinerea*. The early stage included microarray data at 6 hour from GSE5686 and the first two time points from GSE29642. The middle stage of infection was composed of microarray data at 12 hour to 24 hour from GSE5686 and 6 hour to 20 hour from GSE29642. The remaining microarray data were defined as the late stage of infection. A gene was recognized as differentially expressed in a certain infection stage if it was differentially expressed at any of the time points included in this stage. The direction of a differentially expressed gene in each stage was determined by the majority vote of its constituent time points.

### Functional Enrichment Analysis

GO enrichment analyses for modules predicted from MCL, the common response network and the network components were conducted using BiNGO 3.0.2 in Cytoscape with the “GO Biological Process” category^68^. Using the whole annotation as the reference set, overrepresented terms were selected with a significance level of 0.05 (hypergeometric test) after Benjamini-Hochberg false discovery rate correction.

## Additional Information

**How to cite this article**: Jiang, Z. *et al.* Network-Based Comparative Analysis of *Arabidopsis* Immune Responses to *Golovinomyces orontii* and *Botrytis cinerea* Infections. *Sci. Rep.* **6**, 19149; doi: 10.1038/srep19149 (2016).

## Supplementary Material

Supplementary Information

Supplementary Table S1

Supplementary Table S5

## Figures and Tables

**Table 1:** GO enrichment of two major network components.

Components	GO-ID	Corrected *p*-value^a^	Number of associated genes	Description
DefRC	50896	6.09 × 10^−44^	179	Response to stimulus
	6950	8.23 × 10^−26^	109	Response to stress
	9607	8.62 × 10^−21^	53	Response to biotic stimulus
	51707	6.23 × 10^−18^	48	Response to other organism
	6952	5.47 × 10^−13^	45	Defense response
DevRC	15979	1.50 × 10^−18^	21	Photosynthesis
	32501	2.25 × 10^−17^	61	Multicellular Organismal Process
	7275	1.63 × 10^−13^	53	Multicellular Organismal Development
	48856	2.64 × 10^−13^	48	Anatomical Structure Development
	32502	1.06 × 10^−12^	54	Developmental Process

^a^The corrected *p*-values were calculated from the hypergeometric test after Benjamini-Hochberg false discovery rate correction.

**Table 2:** Genes appearing on interactions connecting DevRC and DefRC, which are involved in plant defense and development^a^.

Gene	Symbol	Function in plant development	Function in plant defense
AT4G03190	*AFB1*	Regulates most aspects of auxin responses throughout plant growth and development	Negatively regulates plant defense response to *Hyaloperonospora arabidopsidis* and *P. syringae*
AT4G34460	*AGB1*	Affects multiple developmental processes	*agb1* mutant is more susceptible to *A. brassicicola, B. cinerea, Fusarium oxysporum* and *Plectosphaerella cucumerina*
AT1G59750	*ARF1*	Regulates senescence and floral organ abscission	*arf1* mutant increases resistance against biotrophs
AT5G62000	*ARF2*	Regulates senescence and floral organ abscission	Negatively regulates defense response against *Sclerotinia sclerotiorum*
AT4G02570	*AXR6*	Required for auxin signaling	*axr6* mutant increases susceptibility to *P. cucumerina* and *B. cinerea*
AT1G75080	*BZR1*	Involved in BR-induced growth	Suppresses immune signaling
AT3G51920	*CML9*	Involved in plant growth control	Participates in plant innate immunity
AT1G22920	*CSN5A*	*csn5a* mutant exhibits negative effect on plant development	Targeted by effectors and protected by R proteins
AT1G14920	*GAI*	Represses vegetative growth and floral induction	*gai* mutant promotes susceptibility to virulent *P. syringae* and is more resistant to *A. brassicicola*
AT3G45640	*MPK3*	Regulates stomatal development and patterning	Positively regulates defense response
AT2G43790	*MPK6*	*mpk6* mutant exhibits defect in anther and embryo development	Positively regulates defense response
AT4G35580	*NTL9*	Regulates leaf senescence	Essential for MAMP-triggered stomatal closure
AT1G32230	*RCD1*	*rcd1* mutant displays developmental defects	Participates in regulating balance between plant growth and defense
AT2G01570	*RGA1*	Represses vegetative growth and floral induction	*rag1* mutant shows reduced resistance to *Magnaporthe grisea*
AT4G32570	*TIFY8*	Overexpression of *TIFY8* affects primary root growth	Suppressed by virulent *P. syringae*
AT3G62980	*TIR1*	*tir1* mutant displays diverse developmental defects	Required for susceptibility to *P. syringae*

^a^The corresponding literature reference for each gene is listed in Supplemental Table S6.
