# Supplementary material for: Network-Based Comparative Analysis of Arabidopsis Immune Responses to Golovinomyces orontii and Botrytis cinerea Infections
Source: Sci Rep. 2016 Jan 11;6:19149. doi: 10.1038/srep19149 (PMC4707498; doi:10.1038/srep19149)
Supplement: Supplementary Information [file srep19149-s1.doc]

**Supplemental Information**

**Network-Based Comparative Analysis of *Arabidopsis* Immune Responses to *Golovinomyces orontii* and *Botrytis cinerea* Infections**


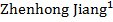
,
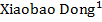
,
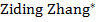


*State Key Laboratory of Agrobiotechnology, College of Biological Sciences, China Agricultural University, Beijing 100193, China*

^1^These authors contributed equally to this work.

* Corresponding author (Email: [zidingzhang@cau.edu.cn](mailto:zidingzhang@cau.edu.cn))

**
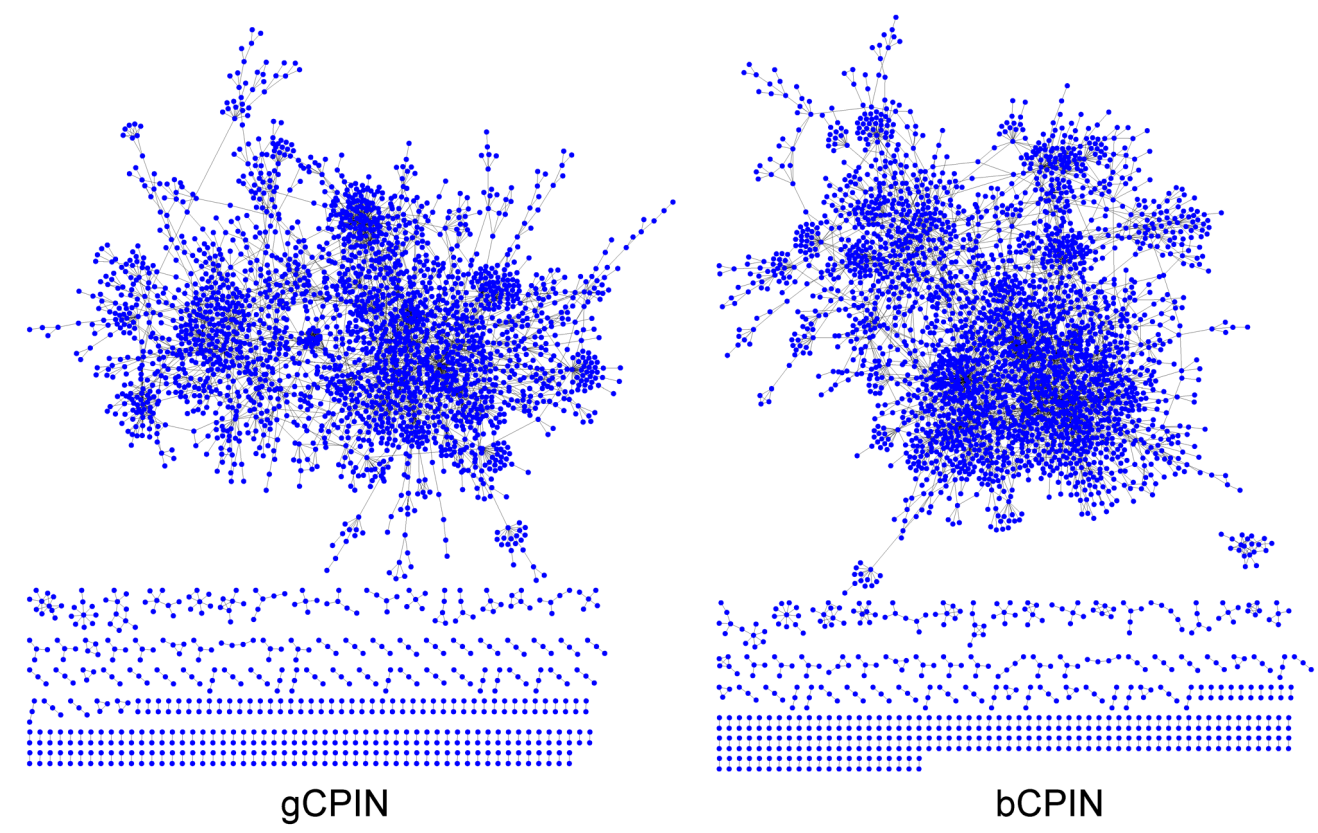
**

Figure S1. **gCPIN and bCPIN are displayed using the Prefuse Force Directed Layout algorithm in Cytoscape.** A node (blue circle) represents a protein, and an interaction (gray line) between two nodes indicates an interaction between two proteins. gCPIN (left) contains 3,101 nodes and 4,353 edges, while bCPIN (right) includes 3,388 nodes and 4,615 edges.

**
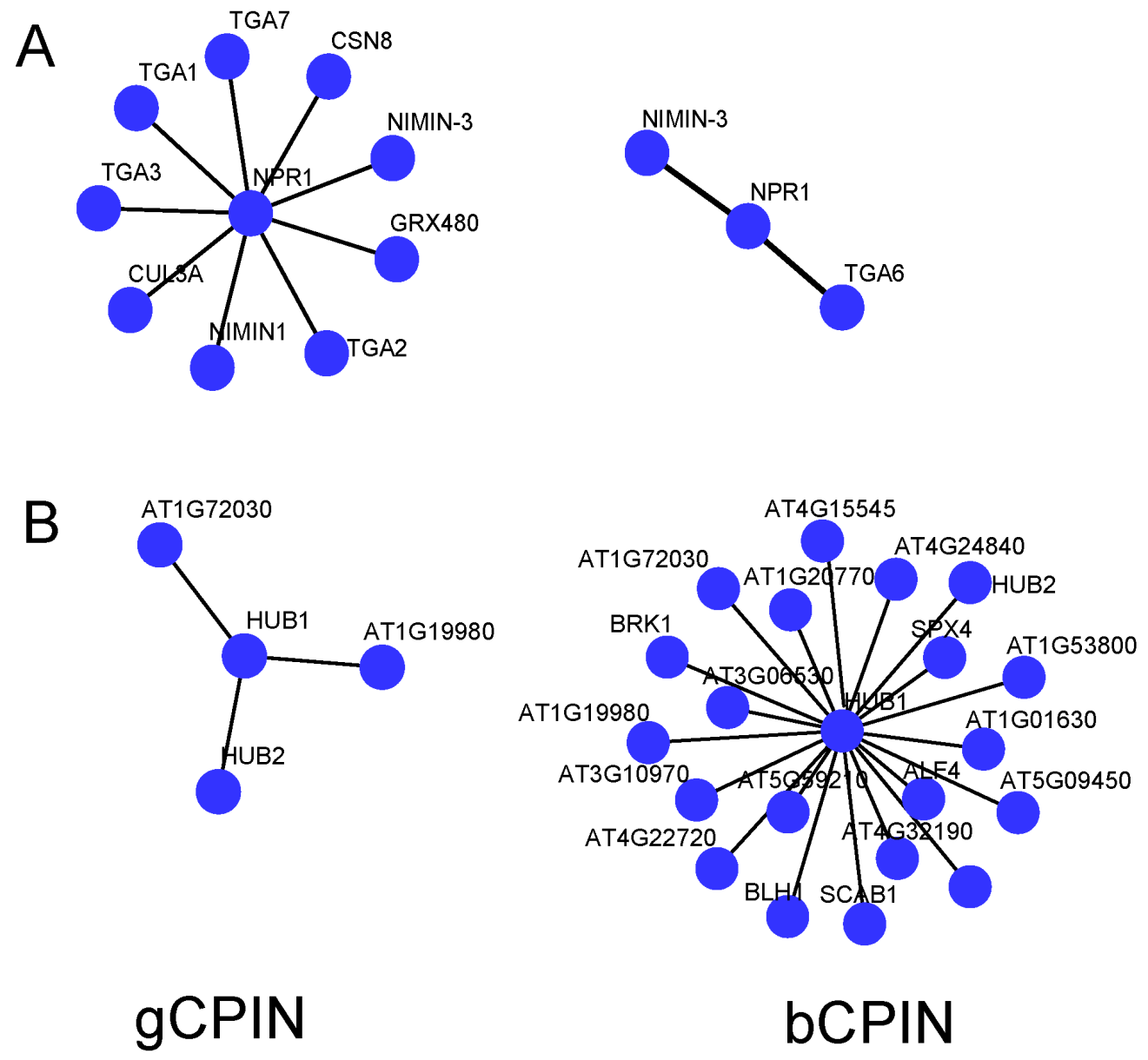
**

Figure S2. **Interaction partners of two hubs in gCPIN and bCPIN**. NPR1 and HUB1 have different numbers of interaction partners in gCPIN and bCPIN. For each node, the corresponding gene symbol is displayed. (A) NPR1 has 9 partners in gCPIN and 2 partners in bCPIN. (B) HUB1 has 3 partners in gCPIN and 20 partners in bCPIN.

**
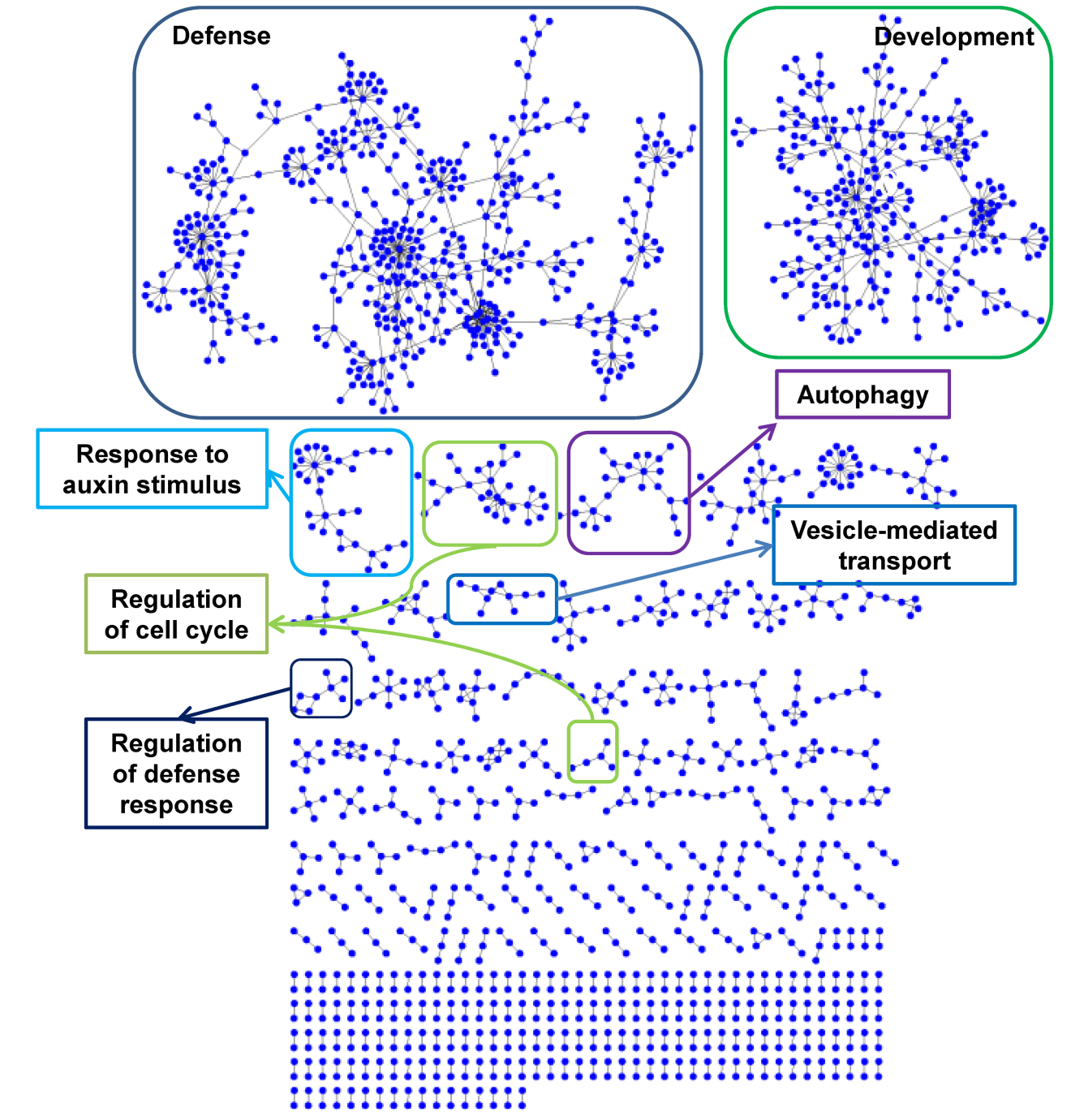
**

Figure S3. **The common response network**. The common response network is displayed using the Prefuse Force Directed Layout algorithm in Cytoscape. An edge between two proteins indicates that this interaction is involved in plant immunity to *G. orontii* as well as *B. cinerea*. The common response network is organized into many connected components. Enriched GO terms for several components are displayed.


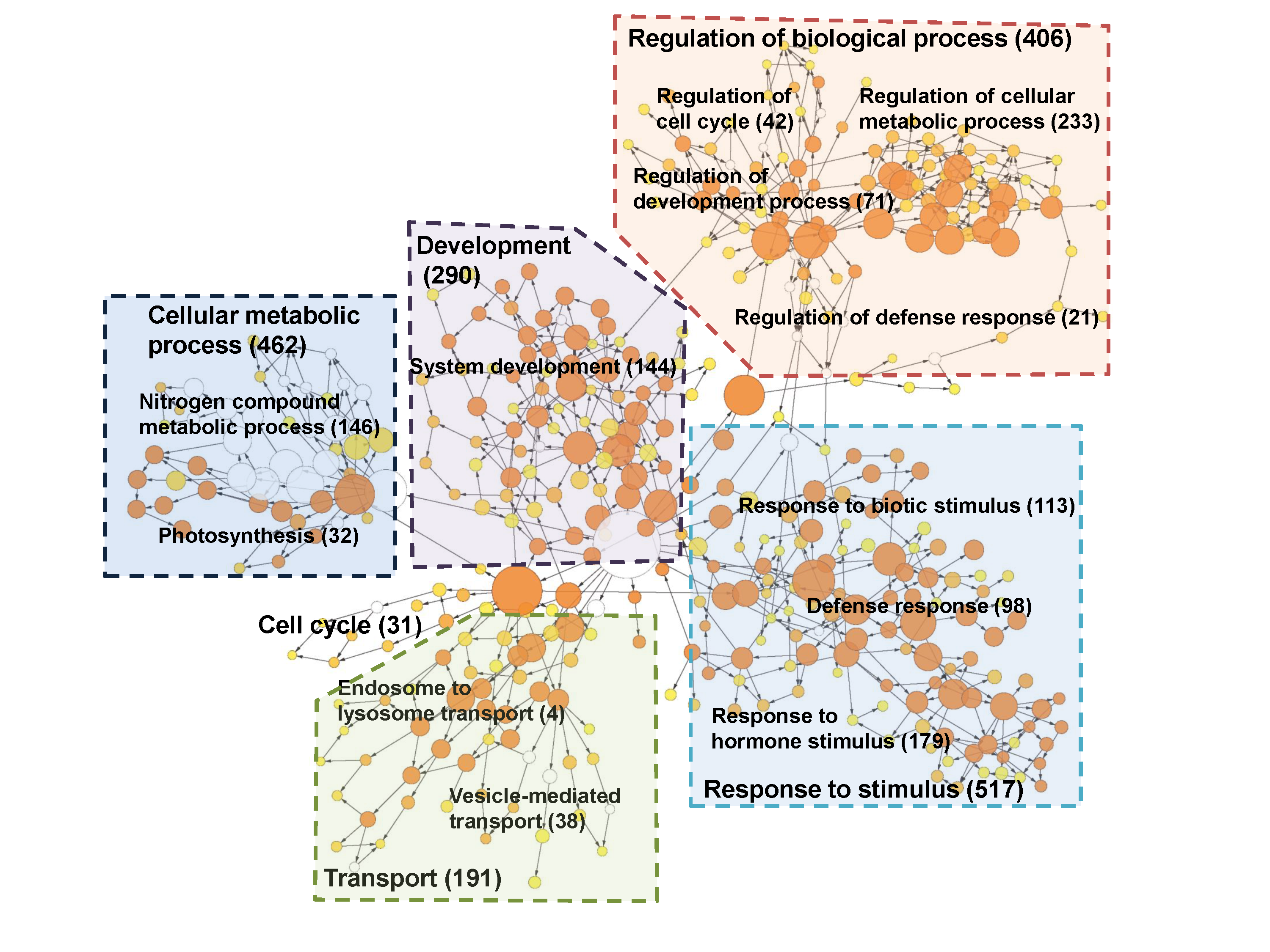


Figure S4. **Annotation results for the common response network.** Annotation analysis was carried out using Cytoscape plugin BiNGO. For better visualization, we chose a significance level of 0.001 instead of the default value 0.05. The annotation results could be divided into six categories, including response to stimulus, development, cellular metabolic process, regulation of biological process, transport and cell cycle. Figures in brackets represent gene number annotated as the corresponding GO term.


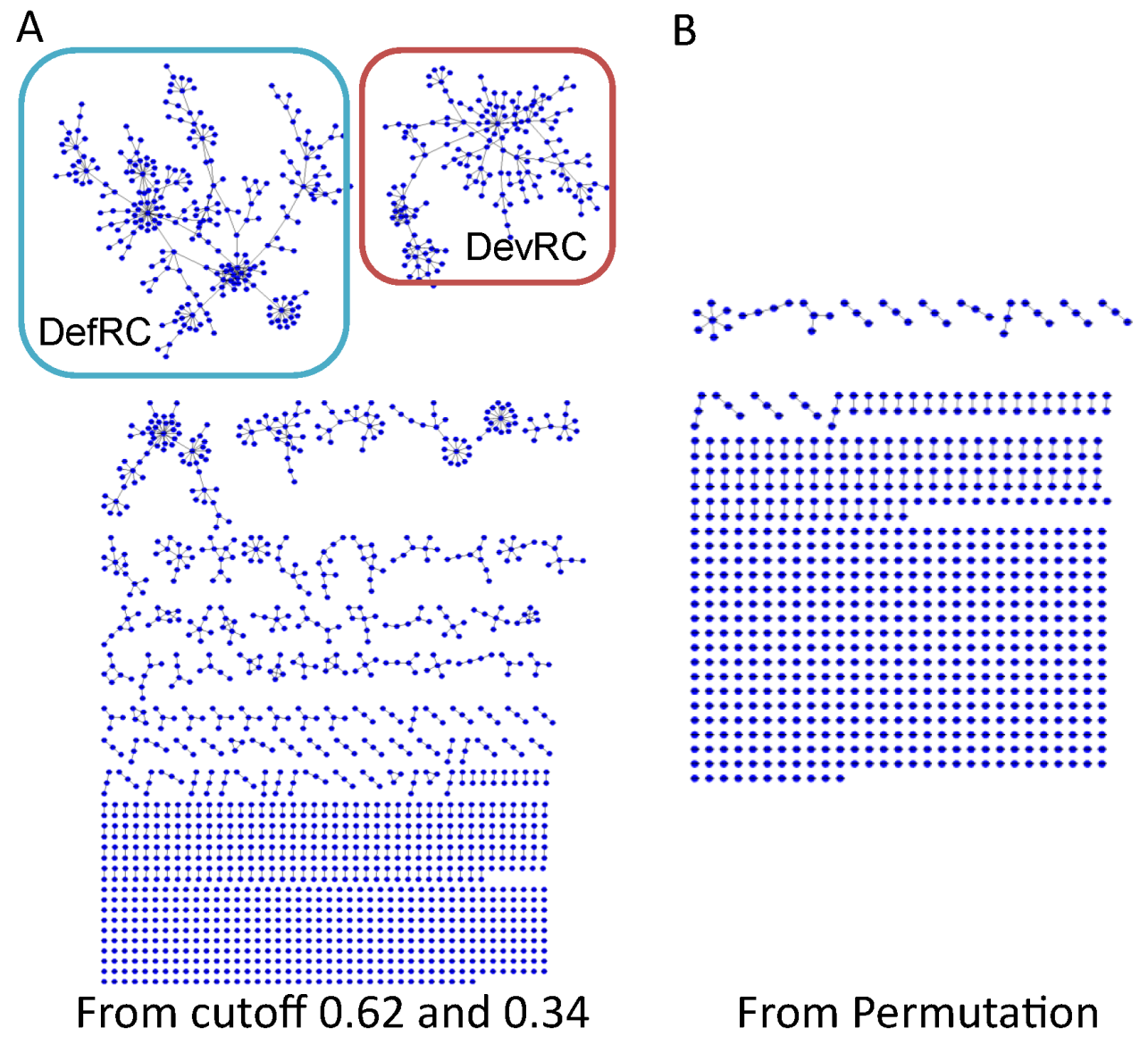


Figure S5. The **common response networks from more stringent PCC cutoff and permutation test.** The common response networks are displayed using the Prefuse Force Directed Layout algorithm in Cytoscape. To obtain a common response network from a more stringent cutoff (A), a PCC threshold (i.e., 0.62 for *G. orontii* and 0.34 for *B. cinerea*, respectively) was selected when the top 5% PCCs from random distributions were treated as correlated. To obtain a common response network from the permutation test (B), transcriptional data were permutated by shuffling expression values for each gene among different time points, and the permutated data were used to construct gCPIN and bCPIN with the PCC threshold of 0.5 and 0.27, respectively.

**
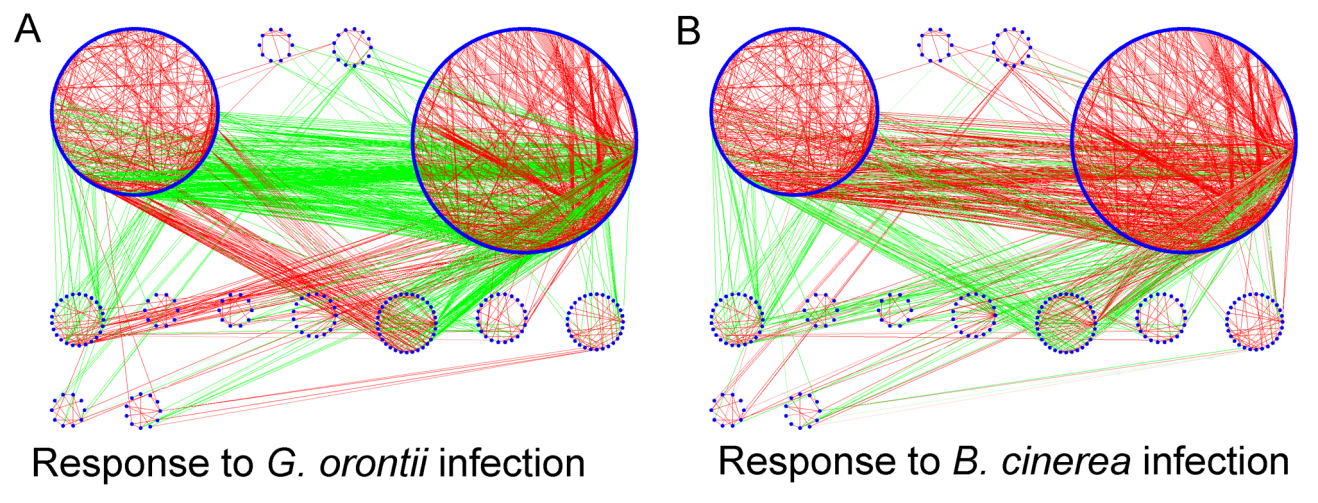
**

Figure S6. **Expression correlation between 13 components.** A red edge represents a positive correlation, and a green edge represents a negative correlation. (A) The expression correlations of interacting protein pairs were calculated using the time-course transcriptional data in response to *G. orontii* infection. (B) Correlations were calculated using the time-course transcriptional data in response to *B. cinerea* infection

.

**
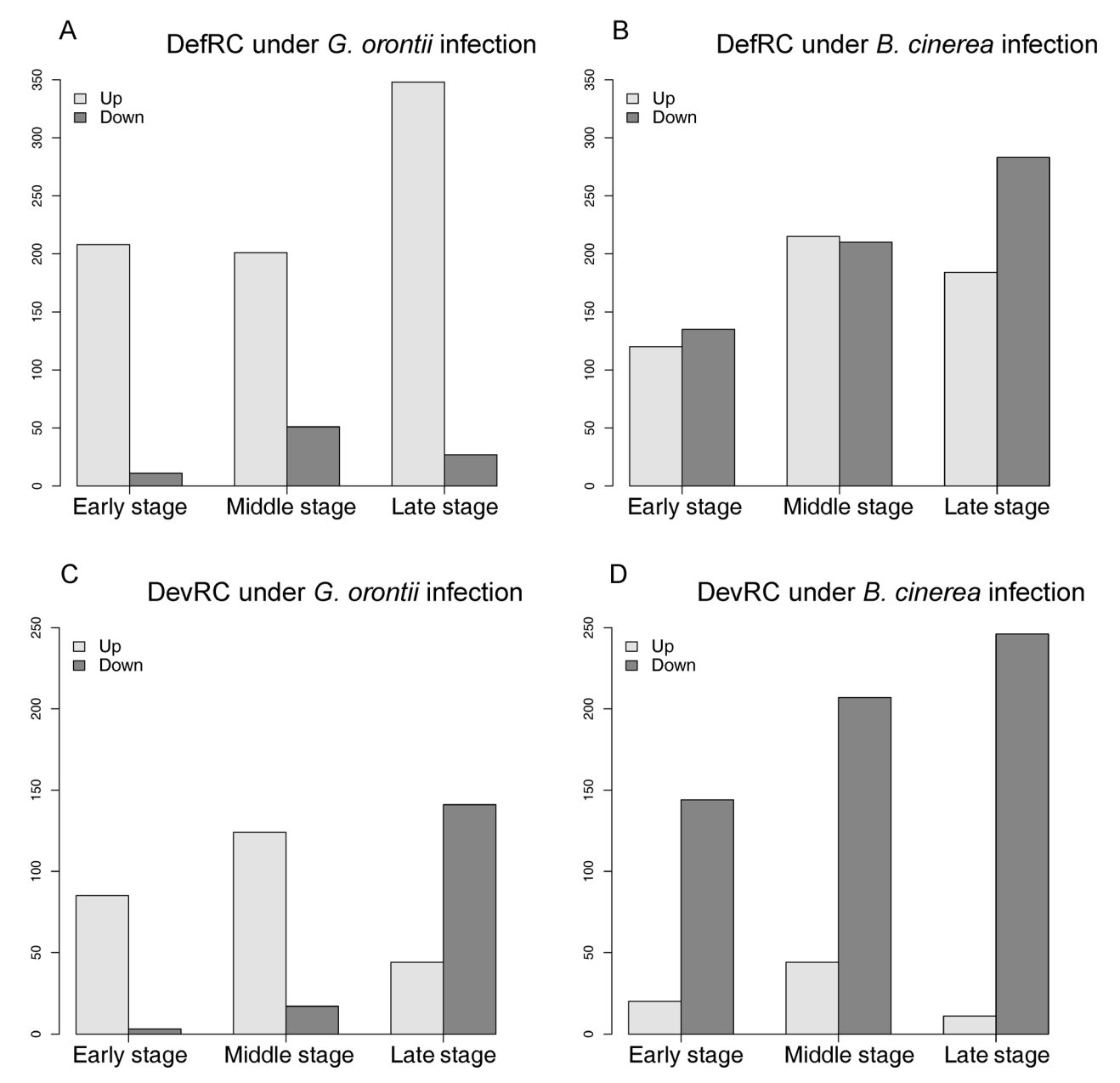
**

Figure S7. **The number of differentially expressed genes in DefRC and DevRC at three infection stages.** Time-course transcriptional data are classified into three stages: early stage, middle stage and late stage.

**
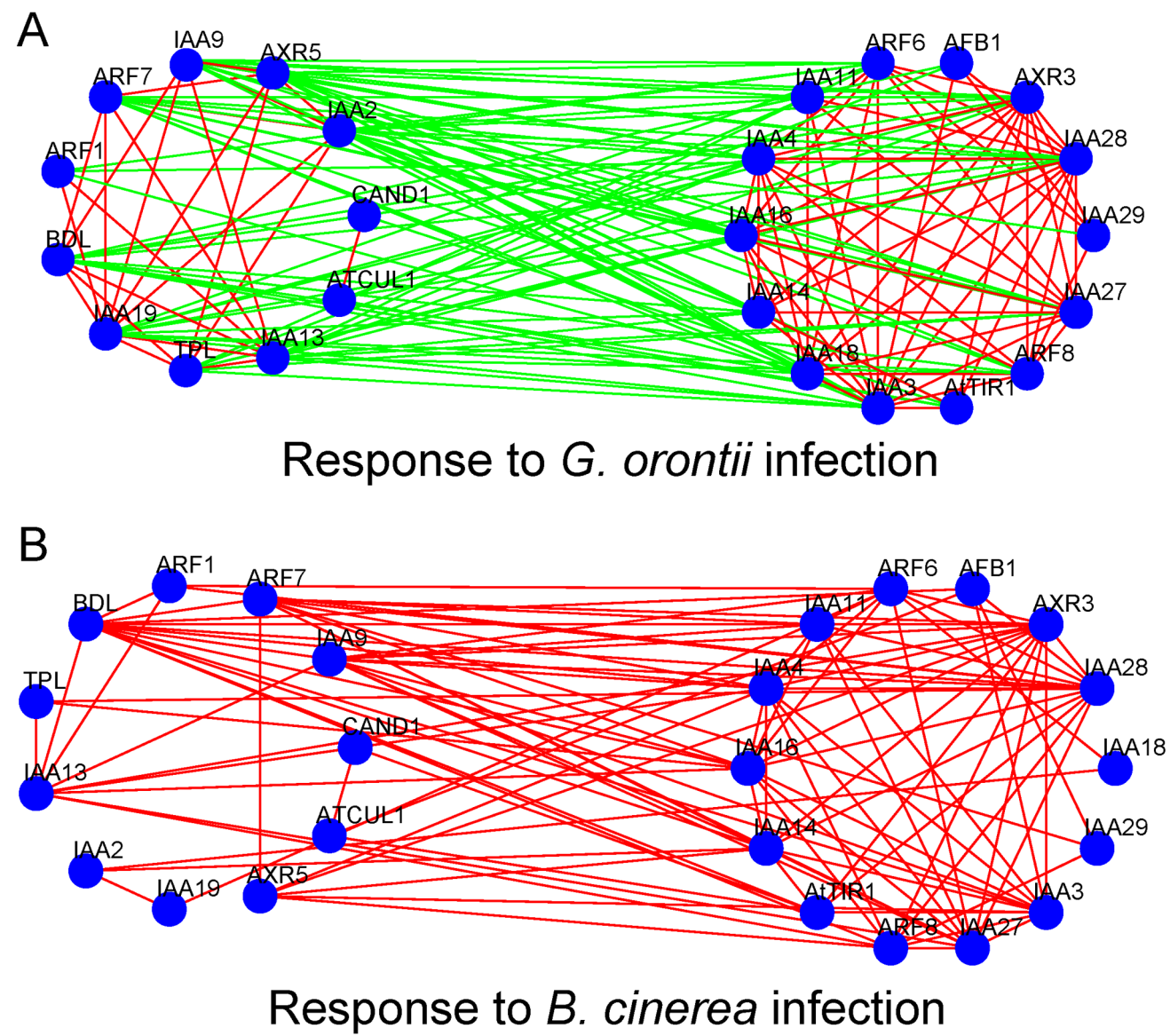
**

Figure S8. **Expression correlation of interactions connecting auxin-related genes from DefRC and DevRC.** Green lines represent negative correlations, and red lines represent positive correlations. The left group denotes auxin-related genes in DefRC, and the right group stands for auxin-related genes in DevRC. (A) All interactions between auxin-related genes from DefRC and DevRC are negatively correlated when responding to *G. orontii* infection. (B) All interactions between auxin-related genes from DefRC and DevRC are positively correlated in response to *B. cinerea* infection.


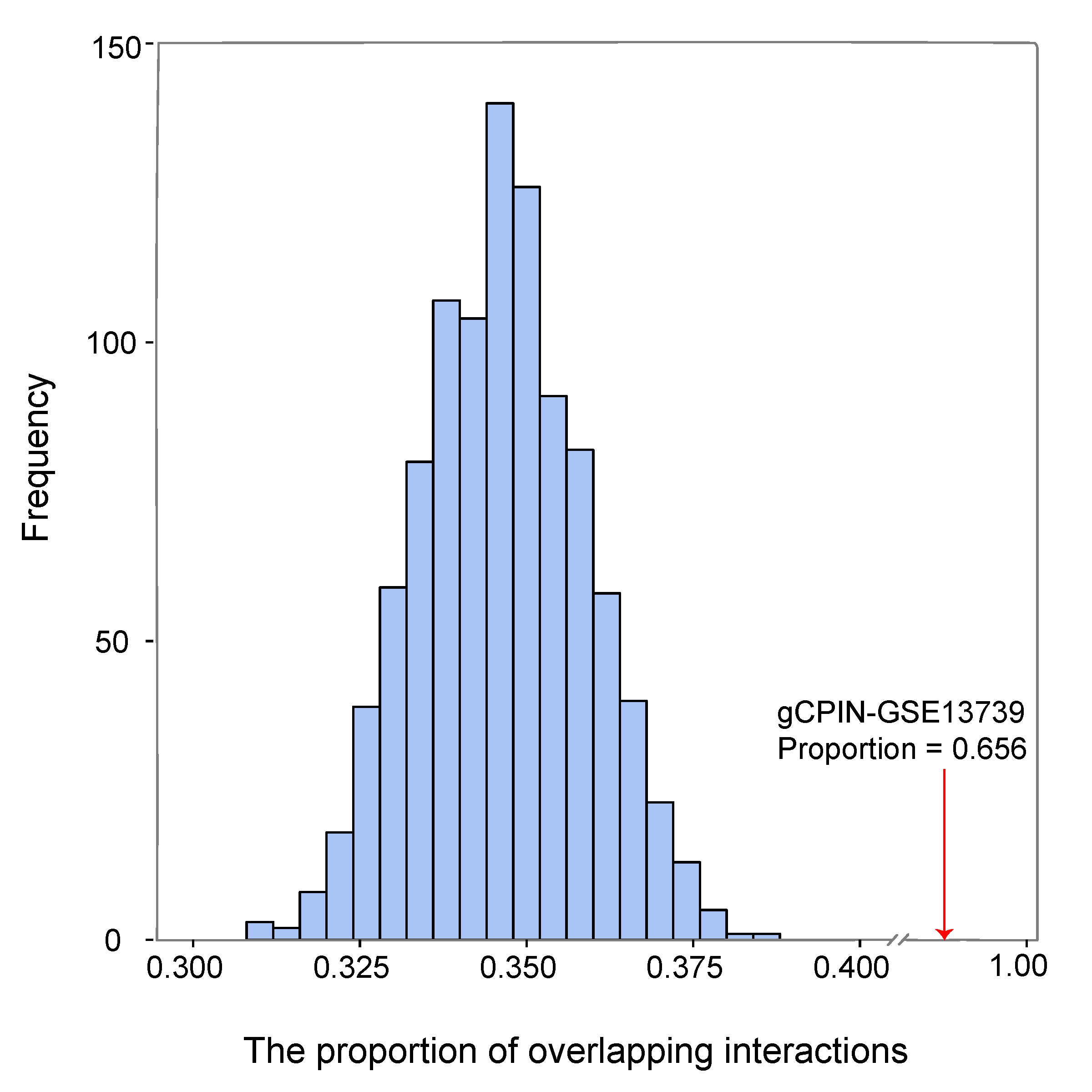


Figure S9. **The distribution of the overlapping interaction proportions between gCPIN and 1000 random conditional networks from permutation experiments.** To obtain 1000 random conditional networks, we permutated the expression data of GSE5686 1000 times and constructed a conditional network for each permutation using the PCC cutoff of 0.5. To construct a new gCPIN (i.e., gCPIN-GSE13739), the raw data of GSE13739 were normalized using RMA from the affy package. To set a significant PCC threshold, we performed the same analyses as described in the main text. Thus, the threshold value of 0.61 was selected for the construction of gCPIN-GSE13739. By keeping interactions with PCC larger than the threshold, the resulting gCPIN-GSE13739 contained 2,754 nodes and 3,454 edges. The proportion of the overlapping interactions between gCPIN and gCPIN-GSE13739 (red arrow) is significantly higher than those between gCPIN and the random conditional networks (Student’s *t* test, *p*-value < 2.2e-16)


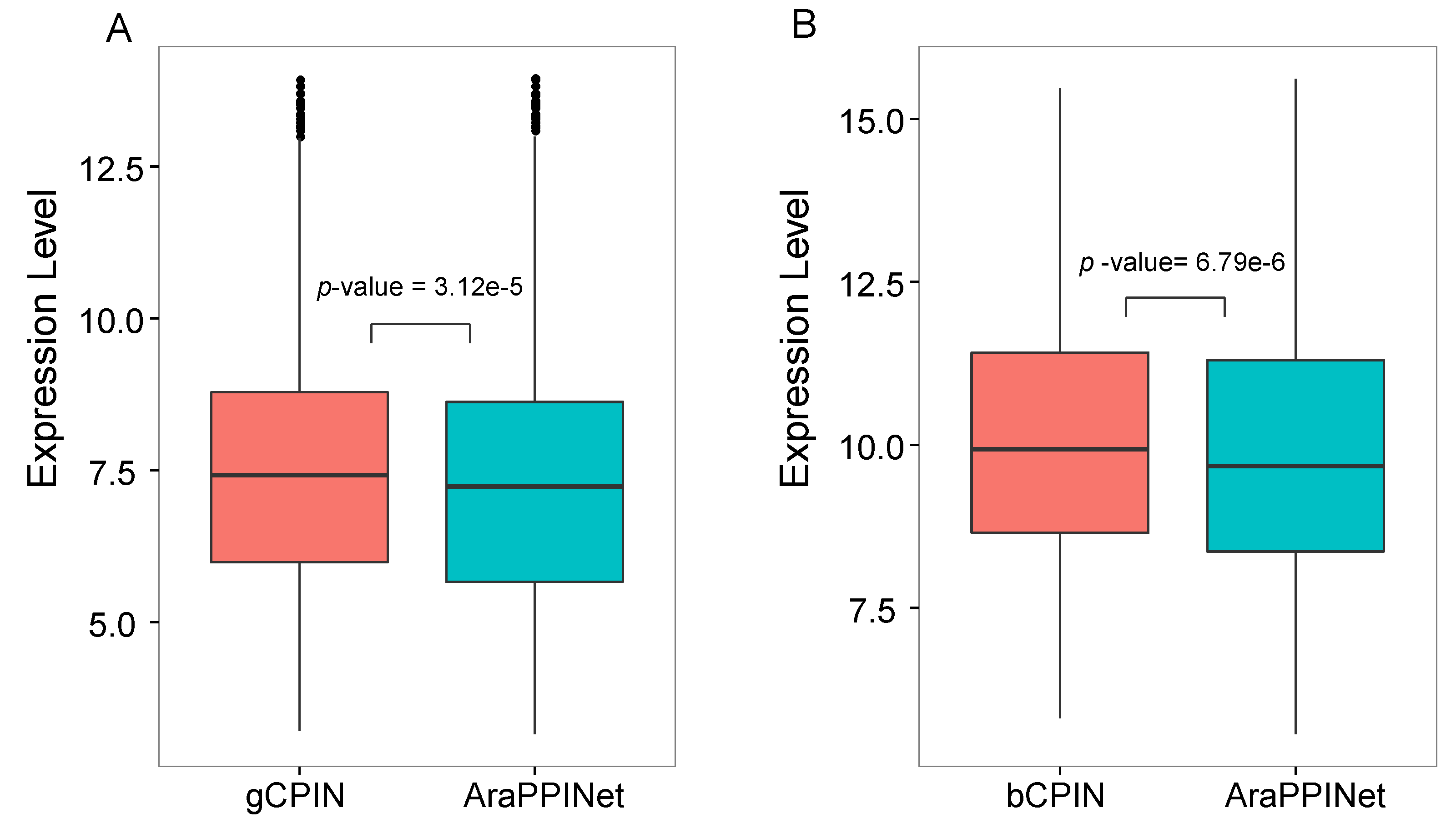


Figure S10**. Comparisons of expression levels for genes in AraPPINet and gCPIN/bCPIN.** The expression levels of genes in the conditional network [gCPIN (A) or bCPIN (B)] were significantly higher than genes in AraPPINet.

**Table S1. Experimentally verified *Arabidopsis* PPI data and PCC values for PPIs under two conditions.** Interaction A and Interaction B represent two proteins in an experimentally verified Arabidopsis PPI. PCC under *G. orontii* (*B. cinerea*) infection condition denote the PCC value calculated using the transcriptional data from tissues infected by *G. orontii* (*B. cinerea*). Label “1” indicates that the corresponding interaction is retained in the conditional network, whereas label “0” indicates that the corresponding interaction is not retained.

*Note that Table S1 is available in a separated Excel file named Table_S1.xls. Due to the large size of this table, we have also made it available at: http://systbio.cau.edu.cn/BN/about.html*

Table S2. Several key network topological features for gCPIN and bCPIN.

|  | Average path length | Average number of neighbors | Clustering coefficient | Connected components | Network diameter | Network density | Network centralization |
| --- | --- | --- | --- | --- | --- | --- | --- |
| gCPIN | 8.733 | 2.807 | 0.079 | 224 | 24 | 0.001 | 0.038 |
| bCPIN | 6.886 | 2.724 | 0.074 | 211 | 19 | 0.001 | 0.057 |

**Table S3. The number of modules annotated as SA-, JA- or ET-related GO terms**.

|  | gCPIN | bPCIN |
| --- | --- | --- |
| JA | 20 | 24 |
| ET | 19 | 26 |
| SA | 18 | 19 |

**Table S4. Hypergeometric test *p*-values** for the enrichment of TF and hormone-related genes in hubs from gCPIN and bCPIN.

|  | gCPIN | bCPIN |
| --- | --- | --- |
| Hormone | 5.55×10^-16^ | 2.62×10^-12^ |
| TF | 1.01×10^-5^ | 5.13×10^-4^ |

**Table S5. Annotation results for two conditional PPI sub-networks, two largest network components and the common response network.**

*Note that Table S5 is available in a separated Excel file named Table_S5.xls. Due to the large size of this table, we have also made it available at: http://systbio.cau.edu.cn/BN/about.html*

**Table S6. The corresponding literature information for each gene listed in Table 2.**

| Gene | Symbol | Function in plant development | Function in plant defense | Reference |
| --- | --- | --- | --- | --- |
| AT4G03190 | *AFB1* | Regulates most aspects of auxin responses throughout plant growth and development | Negatively regulates plant defense response to Hyaloperonospora arabidopsidis and P. syringae | [^1-3^](#_ENREF_1) |
| AT4G34460 | *AGB1* | Affects multiple developmental processes | agb1 mutant is more susceptible to *A.brassicicola, B. cinerea, Fusarium oxysporum* and *P. cucumerina* | [^4-6^](#_ENREF_4) |
| AT1G59750 | *ARF1* | Regulates senescence and floral organ abscission | *arf1* mutant increase resistance against biotrophic pathogens |  |
| AT5G62000 | *ARF2* | Regulates senescence and floral organ abscission | Negative regulates defense response against *Sclerotinia sclerotiorum* |  |
| AT4G02570 | *AXR6* | Required for auxin signaling | *axr6* mutant increase susceptibility to *P. cucumerina* and *B. cinerea* |  |
| AT1G75080 | *BZR1* | Involved in BR-induced growth | Suppress immune signaling |  |
| AT3G51920 | *CML9* | Involved in plan growth control | Participates in plant innate immunity |  |
| AT1G22920 | *CSN5A* | *csn5a* mutant exhibits negative effects on plant development | Targeted by effectors and protected by R proteins |  |
| AT1G14920 | *GAI* | Represses vegetative growth and floral induction | *gai* mutant promotes susceptibility to virulent *P. syringae* and is more resistant to *A. brassicicola*; |  |
| AT3G45640 | *MPK3* | Regulates stomatal development and patterning | Positive regulates defense response |  |
| AT2G43790 | *MPK6* | mpk6 mutant has defects in anther and embryo development | Positive regulates defense response |  |
| AT4G35580 | *NTL9* | Regulate leaf senescence | Essential for MAMP-triggered stomatal closure |  |
| AT1G32230 | *RCD1* | rcd1 mutant displays developmental Defects | Participates in balancing between plant growth and defense |  |
| AT2G01570 | *RGA1* | Repress vegetative growth and floral induction | *rag1* mutant shows reduced resistance to *Magnophorthe grisea* |  |
| AT4G32570 | *TIFY8* | Overexpression of *TIFY8* affects primary root growth | Suppressed by virulent *P. syringae* | [^27^](#_ENREF_27) |
| AT3G62980 | *TIR1* | *tir1* mutant displays diverse developmental defects | Required for susceptibility to *P. syringae* |  |

**Reference**

1. Robert-Seilaniantz, A. *et al.* The microRNA miR393 re-directs secondary metabolite biosynthesis away from camalexin and towards glucosinolates. *Plant J* **67**, 218-231 (2011).

2. Navarro, L. *et al.* A plant miRNA contributes to antibacterial resistance by repressing auxin signaling. *Science* **312**, 436-439 (2006).

3. Dharmasiri, N. *et al.* Plant development is regulated by a family of auxin receptor F box proteins. *Dev. Cell* **9**, 109-119 (2005).

4. Ullah, H. *et al.* The β-subunit of the *Arabidopsis* G protein negatively regulates auxin-induced cell division and affects multiple developmental processes. *Plant Cell* **15**, 393-409 (2003).

5. Llorente, F., Alonso-Blanco, C., Sánchez-Rodriguez, C., Jorda, L. & Molina, A. ERECTA receptor-like kinase and heterotrimeric G protein from *Arabidopsis* are required for resistance to the necrotrophic fungus *Plectosphaerella cucumerina*. *Plant J* **43**, 165-180 (2005).

6. Trusov, Y. *et al.* Heterotrimeric G proteins-mediated resistance to necrotrophic pathogens includes mechanisms independent of salicylic acid-, jasmonic acid/ethylene- and abscisic acid-mediated defense signaling. *Plant J* **58**, 69-81 (2009).

7. Ellis, C. M. *et al.* AUXIN RESPONSE FACTOR1 and AUXIN RESPONSE FACTOR2 regulate senescence and floral organ abscission in *Arabidopsis thaliana*. *Development* **132**, 4563-4574 (2005).

8. Stotz, H. U. *et al.* Jasmonate-dependent and COI1-independent defense responses against *Sclerotinia sclerotiorum* in *Arabidopsis thaliana*: auxin is part of COI1-independent defense signaling. *Plant and Cell Physiology* **52**, 1941-1956 (2011).

9. Llorente, F. *et al.* Repression of the auxin response pathway increases *Arabidopsis* susceptibility to necrotrophic Fungi. *Molecular Plant* **1**, 496-509 (2008).

10. Hellmann, H. *et al.* Arabidopsis AXR6 encodes CUL1 implicating SCF E3 ligases in auxin regulation of embryogenesis. *The EMBO Journal* **22**, 3314-3325 (2003).

11. Lozano-Durán, R. *et al.* The transcriptional regulator BZR1 mediates trade-off between plant innate immunity and growth. *Proc Natl Acad Sci U S A* **109**, 297-302 (2013).

12. Wang, Z.-Y. *et al.* Nuclear-localized BZR1 mediates brassinosteroid-induced growth and feedback suppression of brassinosteroid biosynthesis. *Dev. Cell* **2**, 505-513 (2002).

13. Leba, L.-J. *et al.* CML9, a multifunctional *Arabidopsis thaliana* calmodulin-like protein involved in stress responses and plant growth? *Plant Signaling & Behavior* **7**, 1121-1124 (2012).

14. Leba, L.-J. *et al.* CML9, an *Arabidopsis* calmodulin-like protein, contributes to plant innate immunity through a flagellin-dependent signalling pathway. *Plant J* **71**, 976-989 (2012).

15. Gusmaroli, G., Feng, S. & Deng, X. W. The *Arabidopsis* CSN5A and CSN5B subunits are present in distinct COP9 signalosome complexes, and mutations in their JAMM domains exhibit differential dominant negative effects on development. *Plant Cell* **16**, 2984-3001 (2004).

16. Weßling, R. *et al.* Convergent targeting of a common host protein-network by pathogen effectors from three kingdoms of life. *Cell host & microbe* **16**, 364-375 (2014).

17. King, K. E., Moritz, T. & Harberd, N. P. Gibberellins are not required for normal stem growth in *Arabidopsis thaliana* in the absence of GAI and RGA. *Genetics* **159**, 767-776 (2001).

18. Dill, A. & Sun, T.-p. Synergistic derepression of gibberellin signaling by removing RGA and GAI function in *Arabidopsis thaliana*. *Genetics* **159**, 777-785 (2001).

19. Meng, X. *et al.* Phosphorylation of an ERF transcription factor by *Arabidopsis* MPK3/MPK6 regulates plant defense gene induction and fungal resistance. *Plant Cell* **25**, 1126-1142 (2013).

20. Wang, H. *et al.* Haplo-insufficiency of MPK3 in MPK6 mutant background uncovers a novel function of these two MAPKs in *Arabidopsis* ovule development. *Plant Cell* **20**, 602-613 (2008).

21. Bush, S. M. & Krysan, P. J. Mutational evidence that the Arabidopsis MAP kinase MPK6 is involved in anther, inflorescence, and embryo development. *J. Exp. Bot.* **58**, 2181-2191 (2007).

22. Kim, H. S. *et al.* A NAC transcription factor and SNI1 cooperatively suppress basal pathogen resistance in *Arabidopsis thaliana*. *Nucleic Acids Res.* **40**, 9182-9192 (2012).

23. Hye-Kyung, Y., Sang-Gyu, K., Sun-Young, K. & Chung-Mo, P. Regulation of leaf senescence by NTL9-mediated osmotic stress signaling in *Arabidopsis*. *Molecules and Cells* **25**, 438-445 (2008).

24. Zhu, Y., Du, B., Qian, J., Zou, B. & Hua, J. Disease resistance gene-induced growth inhibition is enhanced by *rcd1* independent of defense activation in *Arabidopsis*. *Plant Physiol.* **161**, 2005-2013 (2013).

25. Brosché, M. *et al.* Transcriptomics and functional genomics of ROS-induced cell death regulation by *RADICAL-INDUCED CELL DEATH1*. *PLoS Genet.* **10**, e1004112 (2014).

26. Suharsono, U. *et al.* The heterotrimeric G protein α subunit acts upstream of the small GTPase Rac in disease resistance of rice. *Proceedings of the National Academy of Sciences of the United States of America* **99**, 13307-13312 (2002).

27. Cuéllar Pérez, A. *et al.* he non-JAZ TIFY protein TIFY8 from *Arabidopsis thaliana* is a transcriptional repressor. *PLoS One* **9**, e84891 (2014).

28. Yu, H. *et al.* Mutations in the TIR1 auxin receptor that increase affinity for Aux/IAA proteins result in auxin hypersensitivity. *Plant Physiol.* **162**, 295-303 (2013).
